# Information structure in Makhuwa: Electrophysiological evidence for a universal processing account

**DOI:** 10.1073/pnas.2315438121

**Published:** 2024-07-19

**Authors:** Rinus G. Verdonschot, Jenneke van der Wal, Ashley Lewis, Birgit Knudsen, Sarah von Grebmer zu Wolfsthurn, Niels O. Schiller, Peter Hagoort

**Affiliations:** ^a^Max Planck Institute for Psycholinguistics, Nijmegen 6525 XD, The Netherlands; ^b^Leiden University Center for Linguistics, Leiden 2311 BD, The Netherlands; ^c^Donders Institute for Brain, Cognition and Behaviour, Centre for Cognitive Neuroimaging, Radboud University, Nijmegen 6500 HB, The Netherlands; ^d^Leiden Institute for Brain and Cognition, Leiden 2333 AK, The Netherlands; ^e^Department of Linguistics and Translation, City University of Hong Kong, Hong Kong 518057, Hong Kong

**Keywords:** information structure, focus, EEG, Bantu language

## Abstract

There is evidence from both behavior and brain activity that the way information is structured, through the use of focus, can up-regulate processing of focused constituents, likely to give prominence to the relevant aspects of the input. This is hypothesized to be universal, regardless of the different ways in which languages encode focus. In order to test this universalist hypothesis, we need to go beyond the more familiar linguistic strategies for marking focus, such as by means of intonation or specific syntactic structures (e.g., it-clefts). Therefore, in this study, we examine Makhuwa-Enahara, a Bantu language spoken in northern Mozambique, which uniquely marks focus through verbal conjugation. The participants were presented with sentences that consisted of either a semantically anomalous constituent or a semantically nonanomalous constituent. Moreover, focus on this particular constituent could be either present or absent. We observed a consistent pattern: Focused information generated a more negative N400 response than the same information in nonfocus position. This demonstrates that regardless of how focus is marked, its consequence seems to result in an upregulation of processing of information that is in focus.

## Significance

In the quest to understand our human language capacity, linguists and neuroscientists aim to find universal patterns. In this study, we recorded electrical brain activity in speakers of Makhuwa-Enahara, a Bantu language spoken in northern Mozambique. We investigated how the marking of focus influences language processing in this language. In contrast to most Indo-European languages Makhuwa-Enahara uniquely marks focus (highlighting the relevant part of the utterance) in the verbal morphology, instead of prosodically. Our findings point toward a universal pattern where focus marking results in an upregulation of focused information, irrespective of how it is linguistically marked. The universality of focus marking is hence not in its linguistic form, but in the processing consequences it has.

When people communicate, they present their information in such a way that it fits with their interlocutors’ current state of mind. That is, the information is structured to indicate what is already given and what is new or contrastive. One important concept in information structure (hereafter IS) is *focus*. The focus of the sentence often provides the new information (1–3), and some argue that this constituent triggers a set of alternatives (4, 5). Focus is frequently observed in question-answer pairs, for example “Who cooked the sauce? FATHER cooked the sauce”: The focused subject “FATHER” provides the new information. In this example, focus is expressed by a pitch accent on the focused subject. However, focus can be expressed by various linguistic means, cross-linguistically, but also within the same language: Next to pitch accent, English can also use a cleft construction such as “It was father who cooked the sauce.” Therefore, IS can affect the prosodic and/or syntactic structure of sentences, as well as their interpretation (6).

Some behavioral studies have suggested that focused information is processed *more deeply* than nonfocused information. For example, Cutler and Fodor (7) showed that in English phonemes are more readily detected when they are in focus (as opposed to when they are not; see ref. 8 for a recent replication). Similarly, Bredart and Modolo (9) using French showed that people are much more likely to notice semantic illusions (i.e., world-knowledge anomalies) when they are in focus. For example, “Cinderella” was seen as less anomalous when it was not in focus (“It was by seven dwarfs_foc_ that *Cinderella was sheltered before marrying her prince.”) than when it was in focus (“It was *Cinderella_foc_ who was sheltered by seven dwarfs before marrying her prince.”). Additionally, in English, Birch and Rayner (10) showed, using eye tracking, that readers were sensitive to focus manipulations while reading sentences. For example, readers make more regressions to focused items and spend more time rereading them. Deeper processing for focused constituents also has been shown using neuro-correlational techniques. For example, Wang et al. (11) used Mandarin WH-questions to elicit focus to specific parts of a sentence while recording EEG. They showed that the detection of semantic incongruencies was influenced by whether the (in)congruent item was in focus or backgrounded. This was shown through a strongly attenuated N400 effect (a negative-going electrophysiological brain response that is maximal about 400 ms after a listener or reader encounters an unexpected word in the language input) for the backgrounded condition and suggests that IS can modulate the depth of semantic analysis. A later study from the same lab (12) showed a similar attenuation for the P600 (a positive-going electrophysiological brain response occurring about 600 ms after a syntactically anomalous word form in the sentence) as a function of focus/background when the syntactic violation was subtle (i.e., number agreement, e.g., “the guests orders*”). These results indicate that the depth of syntactic processing can be modulated by focus such that not all features present in the input are necessarily processed to their full extent.

In a fMRI study on IS using Dutch (13), common activations between a language task (i.e., a sentence judgment task with semantic illusions crossed with focus) and an auditory spatial attention task were found, indicating that focus markers may recruit a domain general attention network which is susceptible to the focus marking characteristics of a language. These results reveal an interplay between attention and language comprehension, with IS playing a pivotal role in recruiting attentional networks to up-regulate processing of focused constituents to ensure that essential information is sufficiently processed.

The aforementioned studies were carried out in English, French, Mandarin, and Dutch, for which intonation typically is the main linguistic strategy to express focus. For these languages, the overarching finding from the literature is that focused constituents are processed more deeply. The question we would like to answer here is whether this *consequence* of focus on language processing (i.e., upregulation of the focused constituent) is identical regardless of *how* focus is expressed in a language. The underlying hypothesis is that despite cross-linguistic variation in linguistic markers of IS, the processing consequences are universal. That is, IS triggers an increased processing depth (i.e., “upregulation”) for the focus constituent. This upregulation, presumably triggered by a focus marker as a relevance signal, leads to more thorough processing either directly, or through the involvement of the attention network in the brain.

To investigate this further, we turn to Makhuwa, spoken in northern Mozambique, which has a very distinct way of marking focus. We conducted an experimental study on this language. Makhuwa is a Bantu language spoken by about 5,8 million people (14). Here, we concentrate on the Enahara variant, spoken on and around Ilha de Moçambique, as the linguistic underpinnings of its focus system are known in detail (15–17). In Makhuwa, focus can be expressed through verbal inflections. In four conjugational categories, there are two forms of the verb in main clauses, called “conjoint” and “disjoint.” The verb forms do not differ in their tense–aspect semantics, but in their relation with the lexical item that follows the verb (15, 16). In other words, what follows the conjoint verb form is in focus, whereas what follows the disjoint verb form is not in focus, as argued by van der Wal (16).^*^ Both forms and their interpretation are illustrated in example (i) (see *SI Appendix* for the linguistic details).

(i) DJ Nthíyáná o-hoó-cá nráma.
  “The woman ate rice.”
CJ Nthíyáná o-c-aalé nramá_FOC_.
  “The woman ate *rice*.” (18)

Makhuwa-Enahara, therefore, provides a unique opportunity to test the hypothesis that the processing of focus is universally similar, with linguistic information in focus being processed more deeply, even when focus is realized in a very different way compared to other languages in which this phenomenon has been tested before. If this hypothesis holds, we expect an attenuated N400 effect when the critical nouns in sentences like in (i) are out-of-focus compared to when they are in focus. For Makhuwa-Enahara, this means that, similar to Wang et al. (11, 12), we predict the N400 effect to be more pronounced with the conjoint verb form than with the disjoint verb form. To test our prediction, we presented target words in sentences with a conjoint verb form (in focus) or disjoint verb form (out of focus), which were either semantically anomalous (e.g., “*I eat bottles*”) or not (e.g., “*I eat rice*”).

## Results

### Behavioral Results.

The mean accuracy to the control questions was 58 ± 11% (range: 39 to 90%; see Table 1 for a breakdown by trial type).

**Table 1.** Behavioral performance on control questions broken down by trial type: Mean (SD) across participants

Trial type	Total trials	Correct trials	Accuracy (%)	Accuracy range (%)
Congruent	33.38 (4.56)	20.82 (5.41)	62 (0.13)	31 to 89
Incongruent	33.14 (3.96)	19.46 (4.49)	59 (0.12)	38 to 94
Fillers	16.16 (2.91)	8.1 (3.13)	50 (0.16)	22 to 95

This number is rather low and is likely due to the fact that our participants had a relatively low educational background (as indicated by our local research assistant) and were unfamiliar with the experimental setting and the metalinguistic requirements that the behavioral task imposes. Additionally, in hindsight, some of the questions we asked might have elicited uncertainty about the correct answer. For example, in the focused incongruous sentence “Emperima tsinca *eshaavi ni ephera” (“Bats eat *keys and guavas”), a possible control question was “*Ninlavula mwaha w’enama*” (“We are talking about animals”), to which participants should have responded Yes (as we were talking about bats). However, some participants might have considered those “bats” not to be real animals as real bats do not eat keys and might have responded No. Since the purpose of the control questions was simply to keep attention on the task, the low accuracy is not a major issue for our study, and we did obtain significant N400 effects indicating successful processing of the target sentences. We cannot however rule out the possibility that the size of the N400 semantic congruency effects in our study may have been reduced by the failure of some participants on some proportion of the trials to notice the semantic anomalies.

### ERP Results.

**Fig. 1.**
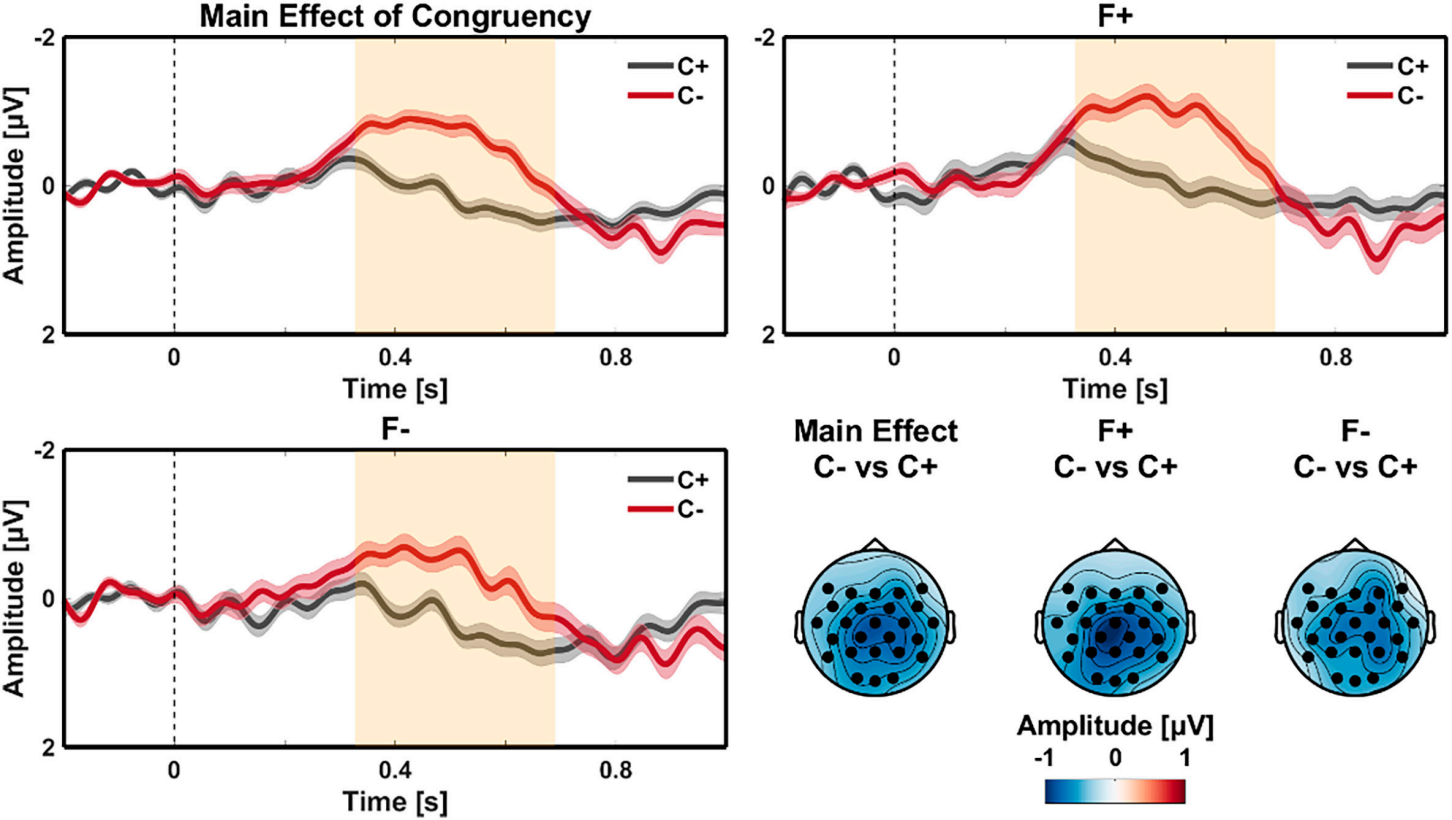
Main effect of Congruency—N400 time window. ERP waveforms of the congruent (C+; black) and incongruent (C−; red) conditions at the critical word (onset at 0 ms) for the main effect of Congruency (*Top Left*), and when the critical word was in focus (F+; *Top Right*) or not in focus (F−; *Bottom Left*). All waveforms diverge from around 330 to 690 ms after the onset of the critical word, and the topographical distribution of the main effect (*Bottom Right*, first topography; centro-parietal maximum) is highly similar to the difference observed when the critical word was in focus (*Bottom Right*, second topography) and when it was not (*Bottom Right*, third topography). Negative is plotted up; shaded regions in the waveforms indicate SEM; orange shaded rectangles indicate time windows corresponding to statistically significant effects; scalp topographies depict the mean amplitude over the time interval corresponding to the effects; The waveforms represent the average of the electrodes that contribute to the N400 effect identified in the cluster-based permutation statistics; black filled circles in the scalp plots indicate electrodes for a statistically significant effect.

The factor Congruency resulted in a negative going ERP effect between 330 and 690 ms after critical word onset. The effect had a centro-parietal maximum (Fig. 1). Based on the timing and topography, we identified this as an N400 congruency effect, with a larger N400 amplitude for the incongruent condition compared to the congruent condition (*P* < 0.001; mean amplitude difference = −0.775 µV).

**Fig. 2.**
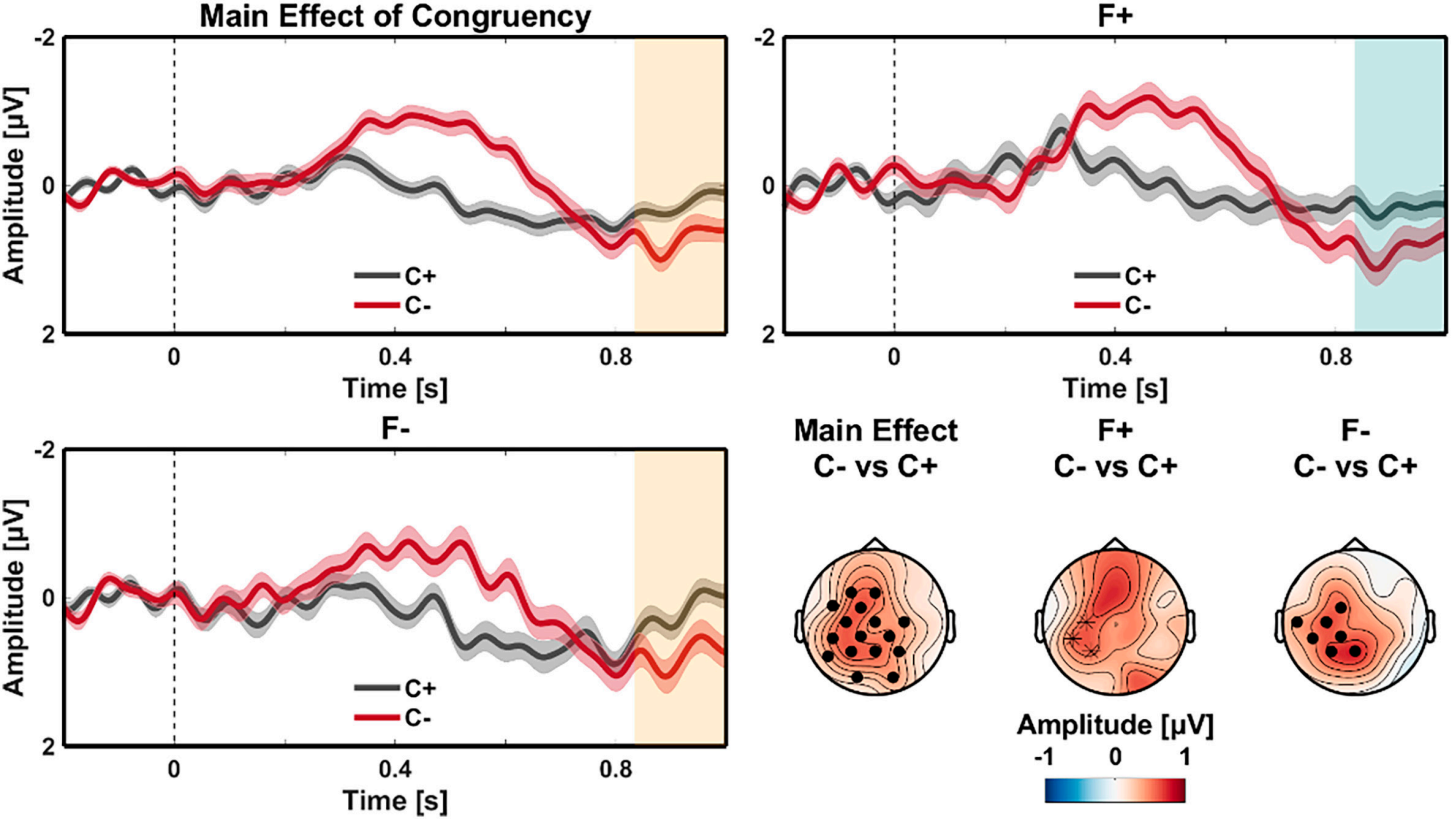
Main effect of Congruency—Post-N400 time window. ERP waveforms of the congruent (C+; black) and incongruent (C−; red) conditions at the critical word (onset at 0 ms) for the main effect of Congruency (*Top Left*), and when the critical word was in focus (F+; *Top Right*) or not in focus (F−; *Bottom Left*). All waveforms diverge from around 835 ms to the end of the time window of interest. Negative is plotted up; shaded regions in the waveforms indicate SEM; orange shaded rectangles indicate time windows corresponding to statistically significant effects; teal shaded rectangles indicate time windows corresponding to marginal effects; scalp topographies depict the mean amplitude over the time interval corresponding to the effects; The waveforms represent the average of the electrodes that contribute to the effect in the cluster-based permutation statistics; black stars in the scalp plots indicate electrodes for a marginal effect—filled circles indicate electrodes for a statistically significant effect.

The N400 effect was followed by a positive going voltage deflection for the main effect of Congruency from 835 ms to 1,000 ms after critical word onset. This effect had a central maximum (see Fig. 2; *P* < 0.05; mean amplitude difference = 0.461 µV), with a slight left hemisphere bias. This effect very likely represents a post-N400 positivity (19).

In addition, a main effect of Focus was observed. The critical words in the focused condition showed a larger negative going voltage deflection than the critical words in the not-in-focus condition. This negativity extended from 285 ms to 690 ms after critical word onset (*P* < 0.025; mean amplitude difference = −0.497 µV). It had a bilateral fronto-central maximum. This topographic distribution was not significantly different from the topography of the N400 Congruency effect (*SI Appendix*).

The Congruency effect was slightly larger in the Focus condition than in the Not-in-Focus condition (−0.811 µV vs. −0.714 µV respectively; see Fig. 1). However, the interaction between Congruency and Focus failed to reach significance. Table 2 summarizes the statistical results. Pairwise comparisons showed that the effects of Congruency and Focus were also (marginally) significant for the separate levels of each factor (Table 2).

**Table 2.** The output of *cluster-based permutation statistics*

Contrast	Negative cluster *P*-value (time window)	Positive cluster *P*-value (time window)
C− vs. C+	0.0002 (0.330 to 0.690 s)	0.0418 (0.835 to 1 s)
F+ vs. F−	0.0026 (0.285 to 0.690 s)	–
Interaction	–	–
F+C− vs. F+C+	–	0.0638 (0.835 to 1 s)
F-C− vs. F-C+	–	0.0328 (0.835 to 1 s)
F+C− vs. F+C+	0.0002 (0.330 to 0.690 s)	–
F-C− vs. F-C+	0.0002 (0.330 to 0.690 s)	–
F+C+ vs. F-C+	0.0256 (0.285 to 0.690 s)	–
F+C− vs. F-C−	0.0058 (0.285 to 0.690 s)	–

*F = focus, C = semantic congruency*. F+ = in focus; F− = not in focus; C+ = semantically congruent; C− = semantically incongruent; brackets indicate time windows (in seconds) corresponding to each effect based on the output of the cluster-based permutation tests—pairwise comparisons were restricted to time windows detected in the main effects.

## Discussion

Previous research has shown, both behaviorally and neuronally, that linguistic information in focus is processed more deeply compared to nonfocused information (7, 9, 11–13). Here, our question was whether such upregulation of the focused constituent is universal regardless of how focus is realized. We investigated this using Makhuwa-Enahara, a Bantu language spoken in Mozambique, where focus is distinctively expressed through verbal conjugation. Despite a somewhat larger Congruency effect in the Focus condition compared to the Not-in-Focus condition, statistically the N400 Congruency effect was similar in both focus conditions. However, importantly, we also found a *general* effect of Focus; that is, *anything following a conjoint (focus) verb form* elicited a more negative ERP than the same information following a disjoint (not-in-focus) verb form.

The first result (i.e., similar N400), at first glance, seems to be incongruous with earlier studies on the role of focus in sentence processing (11, 12) in which out-of-focus conditions typically elicited attenuated ERP Congruency effects compared to in-focus conditions. However, there are two important differences between these and the current study. The first is a methodological difference. Because the Makhuwa verb form already indicates which sentence element is in focus, we could not present the stimuli with a preceding contextual question, as in Wang et al.’s (11, 12) experiments, or else the ERP effect would already appear when hearing the (wrong) verb form. Another difference is that, in contrast to the Wang et al. (9, 10) studies, what followed the disjoint form was not explicitly backgrounded. Nevertheless, in Makhuwa, the distinction between the two verb forms is crucially dependent on focus (15, 16): Only the conjoint and not the disjoint form may be used in object questions or answers, and the disjoint verb form typically expresses a backgrounding of the object (if there is one).”

In line with this interpretation, in their syntactic violation study, Wang et al. (12) *only* found an influence of focus when the violation was subtle (i.e. not for prominent phrase structure violations). A similar case could be made for some of the semantic-illusion studies mentioned in the introduction, in which people tend to miss the subtle semantic anomaly (e.g., Cinderella has nothing to do with seven dwarfs, which violates our world knowledge). The semantic-illusion effect is rather subtle, and one might expect focus to have more “wiggle room” to enhance the N400 effect there. Outright semantic anomalies on the other hand, as in the current study, may perhaps hit one “over the head”—in other words, one could not miss the semantic anomaly in what one was listening to. Consequently, focus may not have provided any additional benefit as attention might have already been captured sufficiently through the semantic anomaly. That is to say, we might have observed a ceiling effect for the N400, which could not be boosted by focus.

**Fig. 3.**
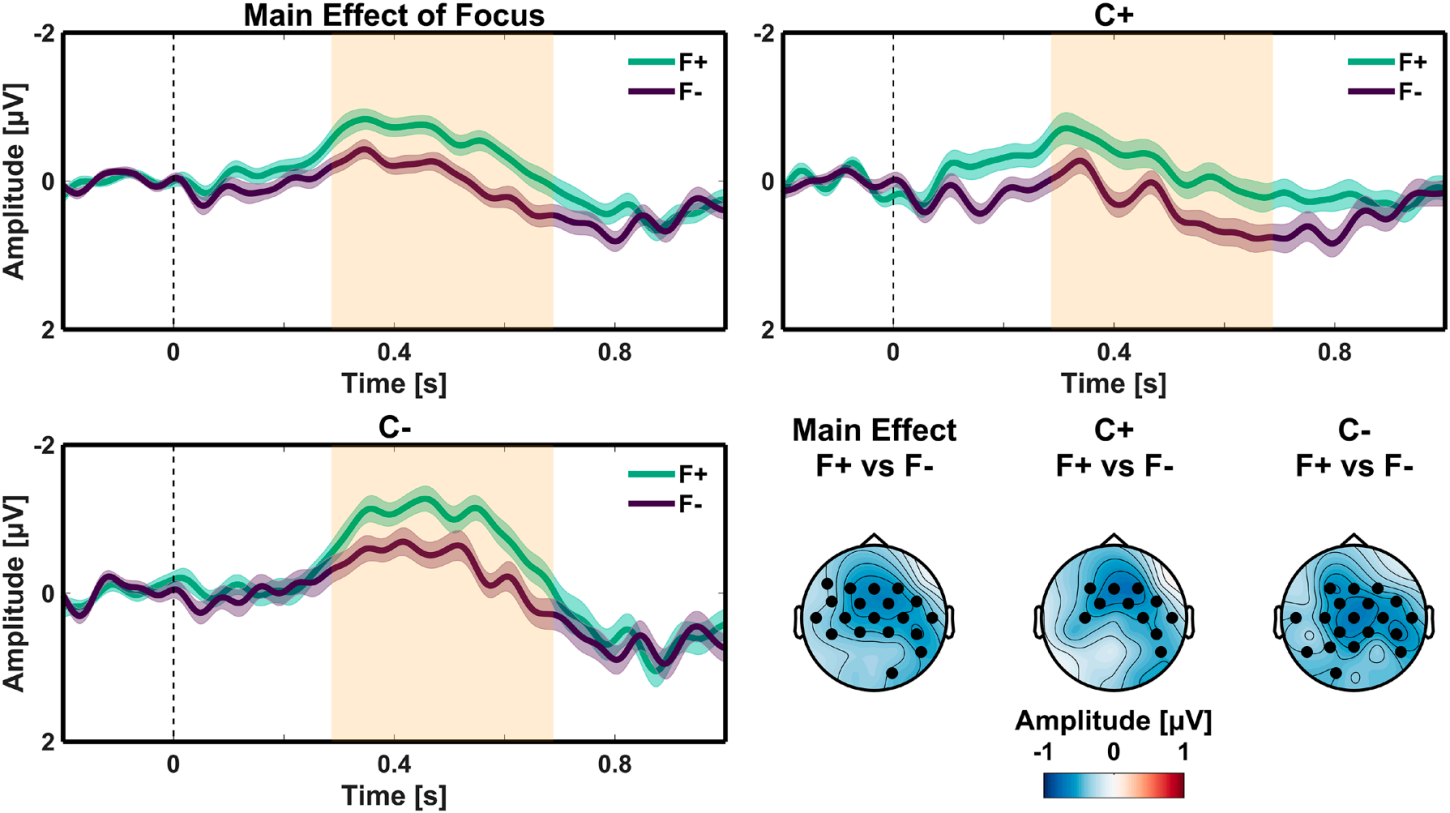
Main effect of Focus. ERP waveforms of the in focus (F+; green) and not in focus (F−; purple) conditions at the critical word (onset at 0 ms) for the main effect of Focus (*Top Left*), and when the critical word was semantically congruent with the preceding sentence context (C+; *Top Right*) or not (C−; *Bottom Left*). All waveforms diverge from around 285 to 690 ms after the onset of the critical word. Negative is plotted up; shaded regions in the waveforms indicate SEM; orange shaded rectangles indicate time windows corresponding to statistically significant effects; scalp topographies depict the mean amplitude over the time interval corresponding to the effects; The waveforms represent the average of the electrodes that contribute to the cluster identified in the cluster-based permutation statistics; black filled circles in the scalp plots indicate electrodes for a statistically significant effect.

However, the most important finding here is that our study indeed revealed an ERP difference in response to the focus of the sentence, wherein nouns following a conjoint (F+) verb form were increasingly more negative compared to their post-disjoint (F−) counterparts. This ERP was present until approximately 700 ms after stimulus onset (as shown in the highlighted regions of Fig. 3), and is, in all likelihood, an N400 effect. This result is also consistent with the hypothesis that a linguistic focus marker results in up-regulating the processing of the focus constituent. In the case of Makhuwa, the conjoint verb form induced the expectation of a focus constituent, resulting in an increased N400 to the following noun.

Overall, the N400 amplitude has been shown to be a sensitive neurophysiological marker of semantic unification (including both anticipation and integration; see refs. 20 and 21). The conjoint verb form as the focus marker in Makhuwa seems to have elicited additional attention, and an increased recruitment of unification operations, to be allocated to the focus constituent. Although this did not result in a significant increase in the N400 effect to a semantic violation (likely due to the aforementioned ceiling effect), the overall stronger N400 to the noun in focus position is strongly indicative of an increased processing effort. In line with earlier studies in languages in which the focus constituent was marked prosodically, and despite the radically different linguistic markers in Makhuwa, its processing consequences seem to be similar, supporting the universalist processing account of linguistic markers of IS.

Finally, we would like to emphasize the importance of investigating lesser-studied languages with diverse linguistic structures. For example, apart from our current study, to the best of our knowledge, only one other EEG study (22) has been conducted on an African (Bantu) language. Yet, due to the complex grammatical structures and vast phonological diversity of these languages, they provide a fertile ground for better understanding the underlying principles of human language in general. Using Makhuwa-Enahara, our study has expanded on the existing body of research on *focus* processing, which has also primarily been conducted using well-studied languages (such as Dutch, English, and Mandarin). By examining languages such as Makhuwa-Enahara, we can advance our knowledge of language acquisition, processing, and representation in the brain, which ultimately helps refine linguistic theories and cognitive models of language processing.

In conclusion, our findings indicate that in Makhuwa-Enahara, information that immediately appears after verb forms indicating postverbal focus (F+) undergoes deeper processing than information that follows nonfocus verb forms (F−). This implies that despite the diverse linguistic means that languages in the world have available to express focus, the impact of focus on language processing is similar. Focus marking results in the upregulation of the information that is in focus. In this way, the speaker can make sure that the new or relevant information is not ignored by the listener.

## Materials and Methods

### Participants.

Fifty-eight healthy right-handed native speakers of Makhuwa-Enahara (48 males, mean age 25.0 ± 6.3) were paid to participate in the experiment. All participants were living on or around Ilha de Moçambique and were recruited through a local research assistant; no neurological abnormalities or hearing deficits were reported. Participants were informed about the procedure in Makhuwa, and all participants signed informed consent (in line with the declaration of Helsinki) before the experiment. Ethics approval was obtained from the Ethics Committee Faculty of Social Sciences at Radboud University, ECSW-2022-056) prior to the start of the study.

### Stimuli.

We created 50 target sentences with four conditions each (totaling 200 sentences) and 88 auditory filler sentences, checked these for naturalness with a native speaker of Makhuwa, and recorded them with the same speaker. These recordings served as the experimental materials (which can be obtained at https://osf.io/qvnpt/). Auditory presentation of the stimuli was chosen as many speakers are not used to reading Makhuwa-Enahara. Two factors were independently manipulated, namely Focus (in focus, not in focus) and Semantic Congruency (congruent, incongruent). For the same sentence, the congruent and the incongruent noun both started with a vowel or they both started with a consonant, enabling the critical epoch selection of the EEG signal to be maximally comparable. Furthermore, the congruent and the incongruent nouns were both object-marked on the verb, or both not object marked, so that these differences are matched between the key contrasts of interest and should not be the cause of any differences observed for those comparisons. Table 3 shows an overview of the four conditions for one of the 50 target sentence quartets. The verb form indicates the focus status: *ki-n-khala* (conjoint) “I stay” indicates focus on the following element, vs. *ki-naa-khala* (disjoint) “I stay” indicates no focus. The critical word is the word directly following the verb; in this example, the incongruent *wiirimu* “heaven” or the congruent *mpaani* “inside.” Note that both these locatives are nouns in Makhuwa.

**Table 3.** Overview of one target set containing four sentences, one for each of the conditions

Condition	Example sentence in Makhuwa (+translation in italics)
Focus: Yes (F+)	Yarupa epula, kinkhala *wiirimu, nkimpheela onaana.
Semantically Incongr. (C−)	*“If it rains, I stay in heaven_foc_, I don’t want to get wet.”*
Focus: Yes (F+)	Yarupa epula, kinkhala mpaani, nkimpheela onaana.
Semantically Congr. (C+)	*“If it rains, I stay inside_foc_, I don’t want to get wet.”*
Focus: No (F−)	Yarupa epula, kinaakhala *wiirimu, nkimpheela onaana.
Semantically Incongr. (C−)	“*If it rains, I stay in heaven*_∅_*, I don’t want to get wet.*”
Focus: No (F−)	Yarupa epula, kinaakhala mpaani, nkimpheela onaana.
Semantically Congr. (C+)	“*If it rains, I stay inside*_∅_*, I don’t want to get wet.*”

F = focus, C = semantic congruency.

The event-related potentials (ERPs) were time-locked to the onset of the critical word immediately following the verb, where the factors Semantic Congruency and/or Focus status were expected to influence sentence processing.

All stimuli were presented to each participant in four blocks (i.e., full within-participant design). Each block contained one of the conditions of the target set (i.e., each block contained one condition of each of the 50 target sets) as well as 22 fillers, and care was taken to distribute each condition equally often in a block. Block order was counterbalanced across participants using a Latin square. Additionally, we created two versions of the experiment in which all items were distributed differently over the blocks using a different Latin square (including different ordering of the fillers). Half of the participants received one version and the other half the other version, but all participants heard all stimuli. As no database exists containing lexical measures (e.g., frequency, etc.) for Makhuwa, we were unable to match lexical characteristics between conditions. The critical word (i.e., the noun immediately following the verb) was never in the sentence-final position.

### Procedure.

Participants were seated in front of a computer monitor at approximately 80 cm distance. All the materials were auditorily presented at a comfortable level via in-ear earphones. A trial consisted of 1) 500 ms blank screen, 2) 1,500 ms fixation cross, 3) auditory stimulus (on the screen an “ear” icon was presented indicating participants had to listen), 4) 1,000 ms blank screen. Then, the next trial started. Additionally, in 33% of the trials (including fillers), a comprehension statement was auditorily presented. Participants needed to answer whether the statement was correct by pressing a green (Yes) or red (No) key on the keyboard. The next trial started after the response. Each participant was instructed in Makhuwa by the local research assistant and began the experiment with a short practice session to familiarize him/her with the experiment. Participants were told that they will be hearing sentences in Makhuwa and that they sometimes will get a question about the sentence they had just heard, which they have to answer by pressing the Yes or the No button. Participants were asked to sit still and relaxed while listening and to try not to blink during a trial.

### EEG Recording and Analysis.

EEG data were recorded using 32 active Ag/AgCl electrodes, 26 of which were mounted in an elastic cap (Acticap, Brain Products, Herrsching, Germany) according to the international 10 to 20 system. Data were referenced online to Fpz (which also acted as the ground). Vertical eye movements and blinks were monitored via a supra- to suborbital bipolar montage and horizontal eye movements using a right-to-left canthal bipolar montage. All electrode impedances were kept below 25 kΩ during the experiment. Recording was done using PyCorder with a sampling frequency of 500 Hz. Eight participants were excluded from the final analyses for the following reasons: The first participant was treated as a pilot participant; four participants were excluded because they did not complete the EEG session; and three participants were excluded due to poor EEG data quality, leaving 43 males and 7 females (age: 24.8 ± 6.3) available for analysis.

All EEG data processing was carried out using the FieldTrip toolbox (23) running in Matlab (R2020b; Mathworks, Inc.). After recording, the data for each participant were rereferenced offline to the average of electrodes placed on the left and right mastoids. A notch filter was applied at 50, 100, and 150 Hz to attenuate any influence of power line noise, and data were high-pass filtered at 0.3 Hz to minimize the influence of slow drift. Data were then epoched from 1,000 ms before to 1,300 ms after the onset of the critical word, and independent component analysis (ICA) was performed on an optimally transformed (for ICA; 1 Hz high-pass filter; DC-offset removed) version of the data. Components capturing eye movements and blinks in the data (24, 25), as well as electrocardiogram (ECG) activity whenever it was observable, were removed from the original untransformed version of the data. An average of 2.5 (SD = 0.93) independent components were removed for each participant. Any remaining epochs containing artifacts were then removed through visual inspection of the data, and bad electrodes (removed prior to ICA) were recovered based on a linear combination of neighboring electrodes.

After artifact rejection, participants’ data were re-epoched from −200 to 1,000 ms relative to critical word onset separately for each experimental condition of interest: In-Focus Semantically Congruent (F+C+); In-Focus Semantically Incongruent (F+C−); Not-In-Focus Semantically Congruent (F-C+); Not-In-Focus Semantically Incongruent (F-C−). On average the following number of trials per participant remained for each condition after artifact rejection: F+C+ (M = 41.82; SD = 3.4); F+C− (M = 41.88; SD = 3.35); F-C+ (M = 42.02; SD = 3.17); F-C− (M = 41.16; SD = 3.53). A low-pass filter was then applied at 30 Hz, and a condition-specific baseline correction was carried out using a baseline period of −200 ms to 0 ms relative to the onset of the critical word. Finally, for each participant epochs were averaged separately within each of the four experimental conditions of interest.

### Statistical Analysis.

For the EEG data, statistical significance was evaluated using cluster-based permutation statistics (26). This procedure involves a paired-samples *t* test performed for every data point comparing the conditions of interest. Any data points not exceeding a preset significance level are discarded (set to zero). Remaining data points are clustered based on their adjacency in space and time, and resultant t-values for all data points in each cluster are summed to produce cluster-level statistics. Participant averages are then randomly assigned to one of the two conditions 10,000 times, each time calculating cluster-level statistics as just described. The permutation distribution is constructed based on the highest cluster-level statistic from each randomization, and statistical significance is assessed by comparing the cluster-level statistics calculated for the measured data against this distribution (cluster corrected *P* < 0.05 was considered significant, and 0.05 < *P* < 0.1 was considered marginally significant).

As a first step, we tested for main effects of Semantic Congruency (C+ vs. C−) and Focus (F+ vs. F−). Next, we tested for an interaction between Semantic Congruency and Focus, taking the mean amplitude in any time windows suggested by the statistical output from the first step, but still forming clusters in space (i.e., across all electrodes). A separate test for this interaction was carried out for each effect detected in the first step. Follow-up tests separately compared F+C− vs. F+C+ and F-C− vs. F-C+ in time ranges corresponding to any observed main effects of Semantic Congruency. Similarly, separate F+C- vs. F-C− and F+C+ vs. F-C+ comparisons were carried out in a time range corresponding to a main effect of Focus.

## Supplementary Material

Appendix 01 (PDF)

## Data Availability

Data have been deposited in ref. 27.

## Footnotes

^*^Note that the disjoint form may but does not need to appear sentence-finally, whereas the conjoint form requires a following constituent.
