# Supplementary material for: Information structure in Makhuwa: Electrophysiological evidence for a universal processing account
Source: Proc Natl Acad Sci U S A. 2024 Jul 19;121(30):e2315438121. doi: 10.1073/pnas.2315438121 (PMC11287159; doi:10.1073/pnas.2315438121)
Supplement: Supplementary file 1 — Appendix 01 (PDF) [file pnas.2315438121.sapp.pdf]

Supplementary Materials: Information structure in Makhuwa: an EEG investigation

S1 Explicit statistical test for topographical differences using linear mixed effects regression.

Visual inspection of the topographies corresponding to the main effect of Congruency suggests a classical centro-parietal N400 distribution, while for the main effect of focus the distribution appears to be maximal over fronto-central electrodes. To explicitly quantify these topographical differences, Bayesian linear mixed-effects regression analyses (Nicenboim & Vasishth, 2016; van de Schoot et al., 2021; Vasishth, Nicenboim, Beckman, Li, & Kong, 2018) were carried out using the *brms* package (Bürkner, 2017) in R (R Core Team, 2020).

We extracted mean amplitude values for every trial in the F+C+, F+C-, F-C+, and F-C- conditions over a time window from 328-688 ms (corresponding to the interval over which the main effects of Congruency and Focus overlap in the cluster-based statistical testing output from the main text), separately for a group of anterior (F3, F7, Fz, F4, F8, FC1, FC5, FC2, FC6) and a group of posterior electrodes (CP1, CP5, CP2, CP6, Pz, P3, P7, P4, P8). A total of 16688 observations (i.e., two amplitude values – one each for the mean over anterior and posterior electrodes respectively – for each participant and every trial after excluding artifacts) were entered into these models. The dependent variable for the models was mean ERP amplitude, and the following fixed effects predictors along with their interactions were included in the models: Congruency (C+ vs C-), Focus (F+ vs F-), Anteriority (anterior vs posterior electrodes). The random-effects structure included intercepts and slopes varying by item and by participant for each of the above predictors. Fixed effects were contrast coded: -0.5 (C-/F+/posterior) and 0.5 (C+/F-/anterior).

We employed regularizing, weakly informative priors (Gelman, Simpson, & Bentacourt, 2017) as follows: intercept parameter and all fixed effects parameters drawn from a normal distribution with mean of 0 and standard deviation (SD) of 1; all random effects parameters and residual error parameters drawn from a truncated (positive only) normal distribution with mean of 0 and SD of 5; all correlation parameters based on LKJ-correlation priors (Lewandowski, Kurowicka, & Joe, 2009) with numerical parameter set to 2 – LKJ(2). Our choice of priors was guided by the knowledge that the ERP data should be roughly centred around zero due to baseline correction, and of typical ERP effect sizes. A prior predictive check was performed.

Model convergence was assessed by inspecting both R statistic values (R-hat) and trace plots for all parameters to ensure that Markov chains were mixing well. Models assumed a linear Gaussian response distribution and empirical posterior estimation for parameters was carried out via the No-U-Turn sampler (Hoffman & Gelman, 2014; Stan Development Team, 2020; 4 separate Markov chains; 10000 sampling iterations per chain, of which 2000 samples were reserved for burn-in) Hamiltonian implementation of Markov chain Monte Carlo (MCMC; Geyer, 1991).

The linear mixed effects modelling confirmed the main effects of Congruency and Focus (Figure S1). As zero falls within the 95% credible interval (CrI) of the posterior estimates for all other fixed effects predictors no clear statistical evidence for our effects appeared being specific to anterior or posterior electrodes. Posterior predictive checks confirmed that the model estimates provide good fits to the data.

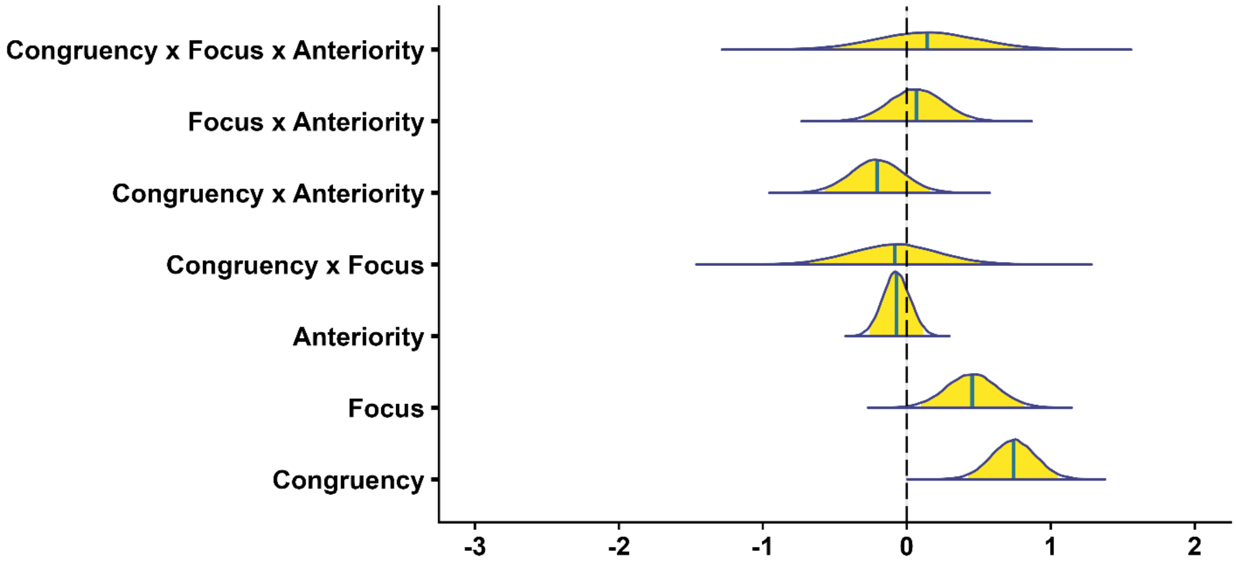

Figure S1. Marginal posterior probability distributions for the fixed effects predictors. Solid vertical blue lines indicate the mean of the distribution and yellow shaded regions indicate 95% credible intervals (CrI), which suggests that the negative main effects of Congruency and Focus are highly reliable (95% CrI does not overlap with zero).

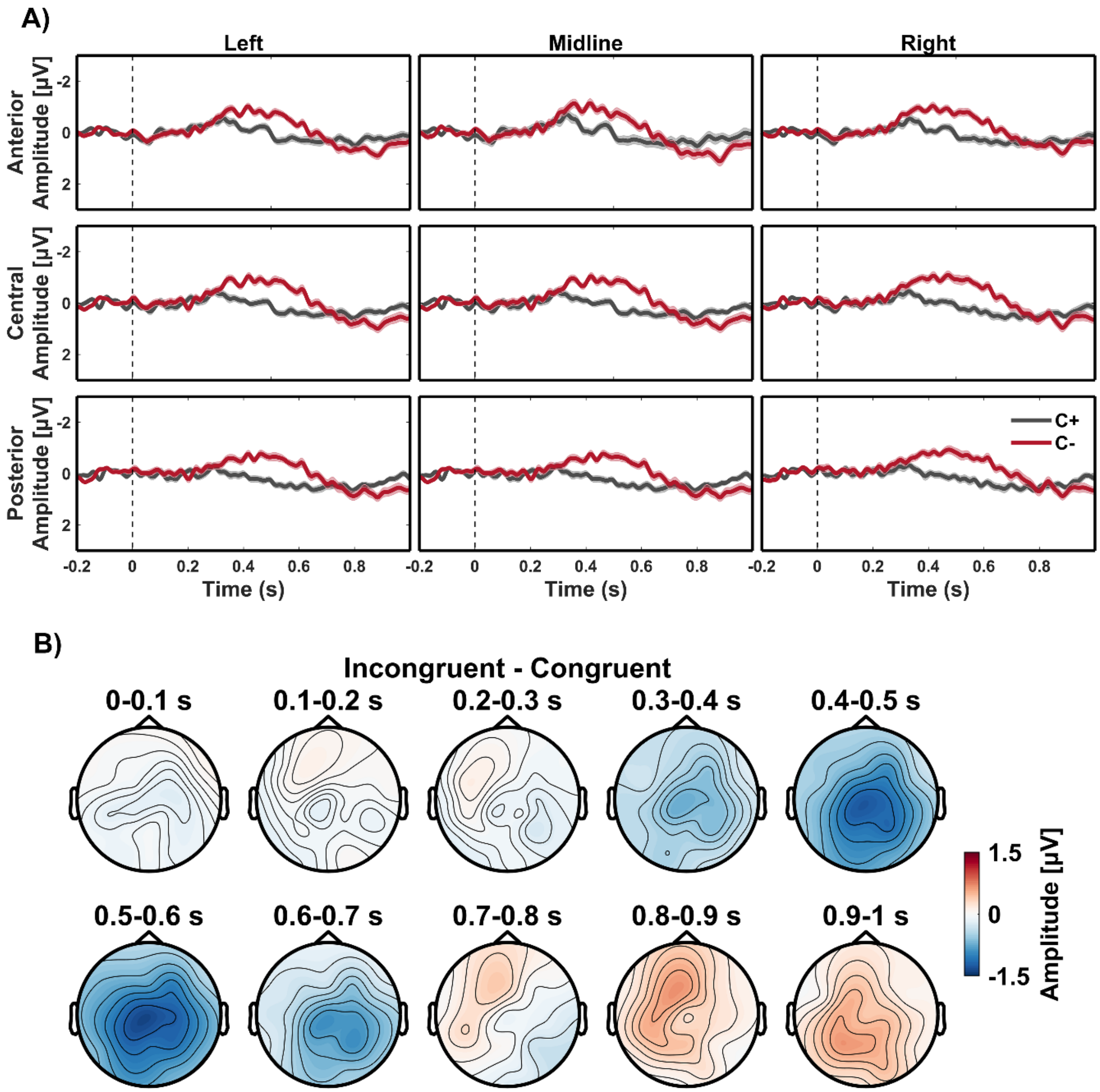

Figure S2. ERP overview for Congruency. (A) ERP waveforms for the C+ (black) and C- (red) conditions at the target word (onset at 0 ms), averaged over electrodes covering different regions of the scalp (Left Anterior: F3, F7, FC1, FC5; Midline Anterior: Fz; Right Anterior: F4, F8, FC2, FC6; Left Central: C3, T7, CP1, CP5; Midline Central: Cz, Pz; Right Central: C4, T8, CP2, CP6; Left Posterior: P3, P7, O1; Midline Posterior: Oz; Right Posterior: P4, P9, O2). Negative is plotted up (following convention); shaded regions indicate standard error of the mean over participants. The waveforms show a divergence between about 350-700 ms that is most pronounced at centro-parietal electrodes, and a later divergence between about 850-1000 ms that is most pronounced at left hemisphere electrodes. (B) Scalp distributions of the difference between conditions (C- – C+) for the mean amplitude over 100 ms time intervals between 0 and 1000 ms after target word onset. The differences mentioned in (A) are clearly visible in the topographies in the 300-400, 400-500, 500-600 and 600-700 ms intervals, and a later difference in the 800-900 and 900-1000 ms interval.

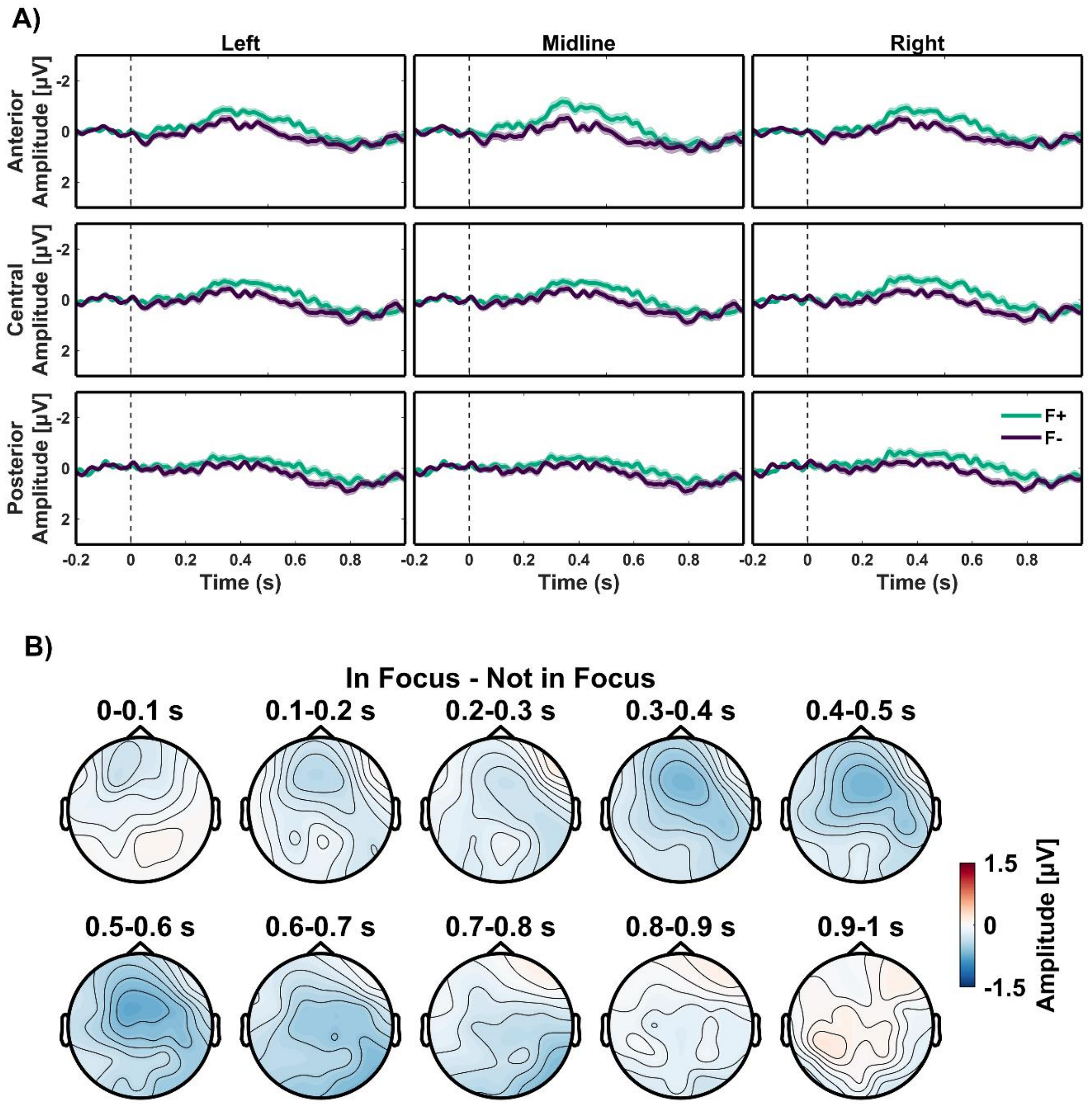

Figure S3. ERP overview for Focus. (A) ERP waveforms for the F+ (green) and F- (purple) conditions at the target word (onset at 0 ms), averaged over electrodes covering different regions of the scalp (Left Anterior: F3, F7, FC1, FC5; Midline Anterior: Fz; Right Anterior: F4, F8, FC2, FC6; Left Central: C3, T7, CP1, CP5; Midline Central: Cz, Pz; Right Central: C4, T8, CP2, CP6; Left Posterior: P3, P7, O1; Midline Posterior: Oz; Right Posterior: P4, P9, O2). Negative is plotted up (following convention); shaded regions indicate standard error of the mean over participants. The waveforms show a divergence between about 300-700 ms that is most pronounced at fronto-central electrodes. (B) Scalp distributions of the difference between conditions (F+ – F-) for the mean amplitude over 100 ms time intervals between 0 and 1000 ms after target word onset. The difference mentioned in (A) is clearly visible in the topographies in the 300-400, 400-500, and 500-600 ms intervals.

S4 Statistical testing for differences beginning before the noun onset.

One possibility is that since the focus marking is carried by inflectional morphology on the verb preceding the target noun in our sentences, there may already be differences in the ERP waveforms between the F+ and F- conditions starting at the verb. Such differences may contaminate the baseline period or may drive the main effect of focus observed after the target noun. To address this potential issue, we carried out a new analysis after changing the baseline period from -0.2 to 0 s relative to noun onset, to 0 to 0.1 s relative to noun onset. Since there were no differences detected in the first 100 ms of the noun time window (the waveforms do not diverge there) in the main analyses, this represents a good baseline window to use for inspecting the waveforms in the preceding time window. We repeated the cluster-based permutation statistics used in the main analyses on the time window that formed the baseline period for those analyses (-0.2 to 0 s relative to noun onset), under the assumption that if there are any differences in this baseline period due to differential focus marking at the verb, those should show up towards the end of the time window in which the verb was processed (i.e., in this 200 ms period directly preceding the noun).

The cluster-based statistical comparison for the main effect of focus (F+ vs F-) did not produce a statistically significant result ($p = 0.75$), and neither did the main effect of congruency (C- vs C+; negative cluster: none; positive cluster: $p = 0.37$), or the interaction (negative cluster: $p = 0.58$; positive cluster: $p = 0.77$). Figure S4 clearly demonstrates that at the electrodes exhibiting a main effect of focus in our main analyses, the waveforms do not diverge in this period that was used as a baseline for those main analyses. We thus conclude that there were no focus effects prior to the onset of the target noun, and so the main effect of focus reflects the influence of focus marking at the verb (in the inflectional morphology on the verb) on the processing of the noun directly following it.

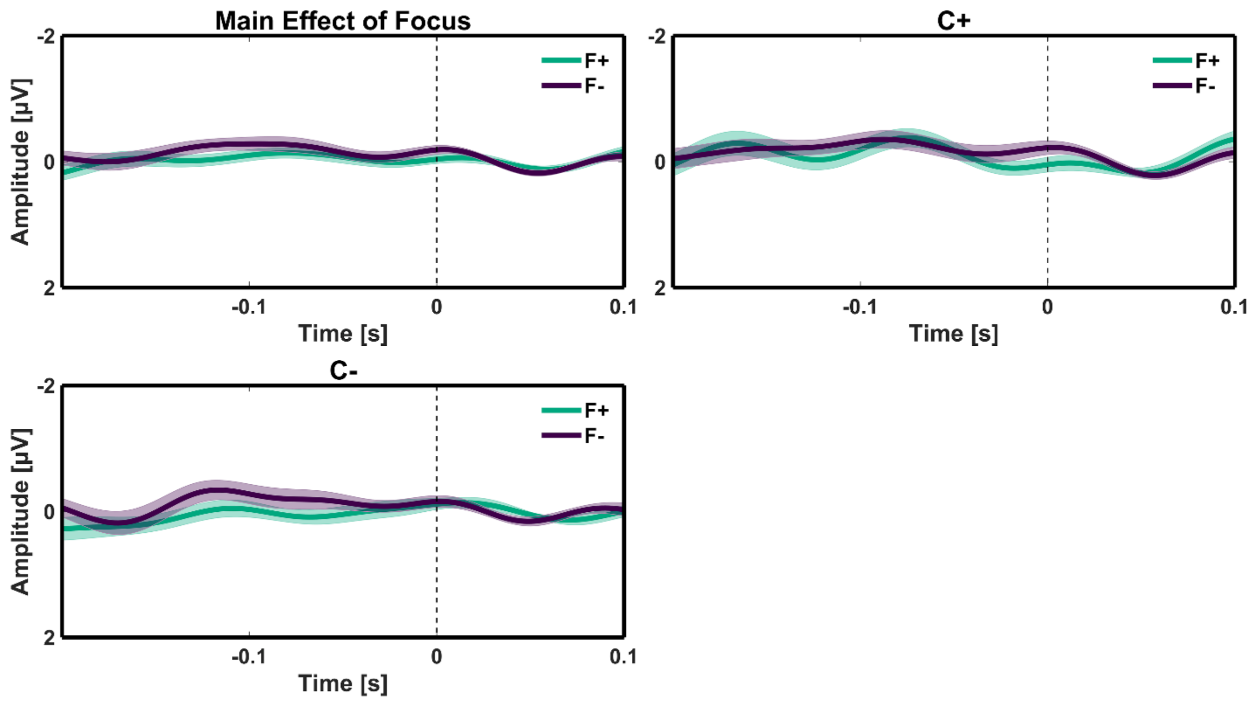

Figure S4. Baseline comparison for main effect of Focus. ERP waveforms of the in focus (F+; green) and not in focus (F-; purple) conditions prior to the critical word (onset at 0 ms) for the main effect of Focus (top left), and when the critical word was semantically congruent with the preceding sentence context (C+; top right) or not (C-; bottom left). The period from 0 to 0.1 s relative to the onset of the critical word (the noun) was used for baseline correction in order to be able to inspect waveforms before the noun onset. There were no statistically significant differences in the period from -0.2 to 0 s based on cluster-based permutation statistics. Negative is plotted up; shaded regions in the waveforms indicate standard error of the mean. The waveforms represent the average of the electrodes that contribute to the first cluster identified in the cluster-based permutation statistics in the main analyses for the main effect of Focus.

Linguistic Details for Example (i)

(i)	DJ	Nthíyáná	o-hoó-cá	nráma.
		1.woman	1SM-PFV.DJ-eat	3.rice
		‘The woman ate rice.’		

	CJ	Nthíyáná	o-c-aalé	nramá$_{\text{FOC}}$.
		1.woman	1SM-eat-PFV.CJ	3.rice
		‘The woman ate *rice*.’ (18)		

Glossary

CJ	Conjoint
DJ	Disjoint
PFV	Perfective
SM	Subject marker

Numbers in the gloss refer to noun classes. In the gloss of a verb form when two numbers are given, the first number represents the subject marker and the second the object marker.

Class	Prefix	Example	Translation
1	N`- / mw-	ńtthu; mwaána	child; person
1a	ø-	totóro; nakhúku	doctor; crow
2	a-	átthu; aána	people, children
2a	á-	ánákhúku	crows
3	N`- / mw-	nvélo; mwaálo	broom; knife
4	mi- / my-	mivélo; myoóno	brooms; arms
5	ni- / n-/	nikútha; naáru; ntáta	knee; ear; hand
6	ma-	makútha; maáru; matáta	knees; ears; hands
9	e-	ekaláwa	dhow
10	e-	ekaláwa	dhows
14	o-	orávo	honey
15	o-	okáttha	to wash
16	va-, wa- (-ni)	vathí; watsulú	on the ground; above
17	o- (-ni)	ontékóni	at work
18	N`- (-ni)	mmáttáni	in the field

Table taken from (van der Wal, 2009)

References

Bürkner, P. C. (2017). brms: An R package for Bayesian multilevel models using Stan. *Journal of Statistical Software*, *80*(1), 1-28.

Gelman, A., Simpson, D., & Betancourt, M. (2017). The prior can often only be understood in the context of the likelihood. *Entropy*, *19*(10), 555.

Geyer, C. J. (1991). Markov chain Monte Carlo maximum likelihood. Manuscript, University of Minnesota.

Hoffman, M. D., & Gelman, A. (2014). The No-U-Turn sampler: adaptively setting path lengths in Hamiltonian Monte Carlo. *J. Mach. Learn. Res.*, *15*(1), 1593-1623.

Lewandowski, D., Kurowicka, D., & Joe, H. (2009). Generating random correlation matrices based on vines and extended onion method. *Journal of Multivariate Analysis, 100*(9), 1989-2001.

Nicenboim, B., & Vasishth, S. (2016). Statistical methods for linguistic research: Foundational Ideas—Part II. *Language and Linguistics Compass*, *10*(11), 591-613.

R Core Team. (2023). *R: A language and environment for statistical computing*. R Foundation for Statistical Computing, Vienna, Austria. Available at https://www.R-project.org/.

van de Schoot, R., Depaoli, S., King, R., Kramer, B., Märtens, K., Tadesse, M. G., Vannucci, M., Gelman, A., Veen, D., Willemsen, J., & Yau, C. (2021). Bayesian statistics and modelling. *Nature Reviews Methods Primers, 1*(1), 1-26.

Vasishth, S., Nicenboim, B., Beckman, M. E., Li, F., & Kong, E. J. (2018). Bayesian data analysis in the phonetic sciences: A tutorial introduction. *Journal of Phonetics*, *71*, 147-161.

Van der Wal, J. (2009). Word order and information structure in Makhuwa-Enahara. Utrecht: LOT.
